# Comprehensive comparison between geriatric and nongeriatric patients with trauma

**DOI:** 10.1097/MD.0000000000028913

**Published:** 2022-02-18

**Authors:** Pei-Chen Lin, Nan-Chun Wu, Hsiu-Chen Su, Chien-Chin Hsu, Kuo-Tai Chen

**Affiliations:** a Graduate Institute of Biomedical Informatics, College of Medicine Science and Technology, Taipei Medical University, Taipei, Taiwan; b Emergency Department, Taoyuan General Hospital, Ministry of Health and Welfare, Taoyuan, Taiwan; c Division of Traumatology, Department of Surgery, Chi-Mei Medical Center, Tainan, Taiwan; d Emergency Department, Chi-Mei Medical Center, Tainan, Taiwan; e Department of Biotechnology, Southern Tainan University of Technology, Tainan, Taiwan; f Department of Emergency Medicine, School of Medicine, College of Medicine, Taipei Medical University, Taipei, Taiwan.

**Keywords:** elderly, geriatric, incidence, outcome, trauma

## Abstract

The incidence of geriatric trauma is increasing due to the growing elderly population. Healthcare providers require a global perspective to differentiate critical factors that might alter patients’ prognosis.

We retrospectively reviewed all adult patients admitted to a trauma center during a 4-year period. We identified 655 adult trauma patients aged from 18 to 64 (nongeriatric group) and 273 trauma patients ≥65 years (geriatric group). Clinical data were collected and compared between the 2 groups.

The geriatric group had a higher incidence of trauma and higher Injury Severity Scores than did the nongeriatric group. Fewer geriatric patients underwent surgical treatment (all patients: geriatric vs nongeriatric: 65.9% vs 70.7%; patients with severe trauma: geriatric vs nongeriatric: 27.6% vs 44.5%). Regarding prognosis, the geriatric group exhibited higher mortality rate and less need for long-term care (geriatric vs nongeriatric: mortality: 5.5% vs 1.8%; long-term care: 2.2% vs 5.0%).

We observed that geriatric patients had higher trauma incidence and higher trauma mortality rate. Aging is a definite predictor of poor outcomes for trauma patients. Limited physiological reserves and preference for less aggressive treatment might be the main reasons for poor outcomes in elderly individuals.

## 1 Background

Geriatric trauma is becoming a major problem because geriatric citizens are projected to constitute approximately one-fifth of the world's population by 2050 and because optimal management strategies for geriatric patients with trauma remain to be determined.^[^1^,^^2^^]^ Elderly patients usually exhibit diverse comorbidities, receive polypharmacy, and have limited physiological reserves, implying a relatively high risk of death and severe disability.^[3]^ A Spanish survey demonstrated that patients aged >65 years accounted for up to 20% of patients admitted to intensive care units.^[4]^ Several studies have focused on the differences between geriatric and nongeriatric patients with trauma. The scopes of such studies included emergency medical services, triage, trauma team activation, trauma mechanisms, thoracic injury, traumatic brain injury, and laboratory tests, in addition to the effectiveness of various trauma scores in a geriatric population and in-hospital management of geriatric patients with trauma.^[^1^,^^5^^–^16^]^

However, the limitation of these studies is that they have only partially addressed geriatric trauma. Healthcare providers require a global perspective to differentiate major factors that might impact a patient's prognosis and the relationship between these factors. Accordingly, we conducted this study to collect comprehensive data, including prehospital records and the entire hospital courses, regarding patients with trauma admitted to a trauma center in southern Taiwan. This study can provide exhaustive information regarding the differences in clinical details between geriatric and nongeriatric patients with trauma.

## 2 Methods

### 2.1 Study patients and data collection

We retrospectively reviewed all patients admitted to the adult wards of a trauma center in southern Taiwan between July 1 and October 31, 2016. This trauma center owns 1288 beds, including 117 beds for intensive care. The estimated numbers of emergency department (ED) visit and hospital admission are 120,000 and 53,000 annually. The identification of a patient with trauma was based on the classification in triage of ED. Of a total of 8297 patients, we excluded 7293 patients without trauma and 76 patients with trauma who were aged <18 years. The remaining 928 patients constituted our study cohort, of whom 655 patients were aged <65 years (nongeriatric group) and 273 patients were ≥65 years (geriatric group), respectively. Trauma incidence was estimated using the census data in the same year. We stratified the geriatric group according to a 10-year interval to observe the trend of interval changes.

We collected the following data: demographic characteristics (age, sex, and Charlson comorbidity index), prehospital presentation (location of injury, mechanism of injury, and time of transportation from the site of injury to the hospital), clinical presentation at ED [vital signs, shock index [heart rate/systolic arterial pressure], triage (Taiwan Triage and Acuity Scale, Taiwan Triage and Acuity Scale),^[17]^ Glasgow Coma Scale, interventions performed at ED, time from the ED to the ward, and time from the ED to the operating room, various trauma scores (Abbreviated Injury Scale score, Injury Severity Score, New Injury Severity Score, Revised Trauma Score, and Trauma Injury Severity Score), hospital course (need for surgery, need for intensive care, hospital length of stay, and intensive care unit length of stay), and prognosis (recovery, mortality, need for long-term care, acute transfer, and complications).

### 2.2 Comparisons between geriatric and nongeriatric group

We compared the data between the geriatric and nongeriatric groups. Because most of the patients included in this study sustained mild injuries and may not represent the actual conditions of severely wounded patients, we conducted a subgroup analysis to address this concern; from all patients with trauma, we selected patients with severe trauma (defined as Injury Severity Score >15) and divided them into geriatric and nongeriatric groups. Subsequently, we compared the collected data regarding the aforementioned variables between these 2 groups of patients with severe trauma.

Moreover, we collected the data of every patient who had undergone surgery during admission and analyzed the outcomes, Injury Severity Scores, and need for intensive care and life-saving procedures. The Institutional Review Board of Human Research, Chi-Mei Medical Center granted this study exemption from approval because the researchers used deidentified data. The study was conducted in accordance with the Declaration of Helsinki.

### 2.3 Statistical analysis

Statistical analyses were performed using SPSS 15 (SPSS, Inc., Chicago, IL). We employed the Chi-Squared test and Student *t* test to evaluate differences in dichotomous and continuous variables, respectively, between the various groups. Continuous data are presented as mean ± standard deviation. Because the time period from injury to the ED, from the ED to the ward, and from the ED to the operating room involved a relatively high number of outliers, we compared these data between the groups by using the Mann–Whitney *U* test; these data are also presented as median and interquartile range. Overall, statistical significance was set at a *P* value of <.05.

## 3 Results

### 3.1 All patients with trauma

**Figure 1:**
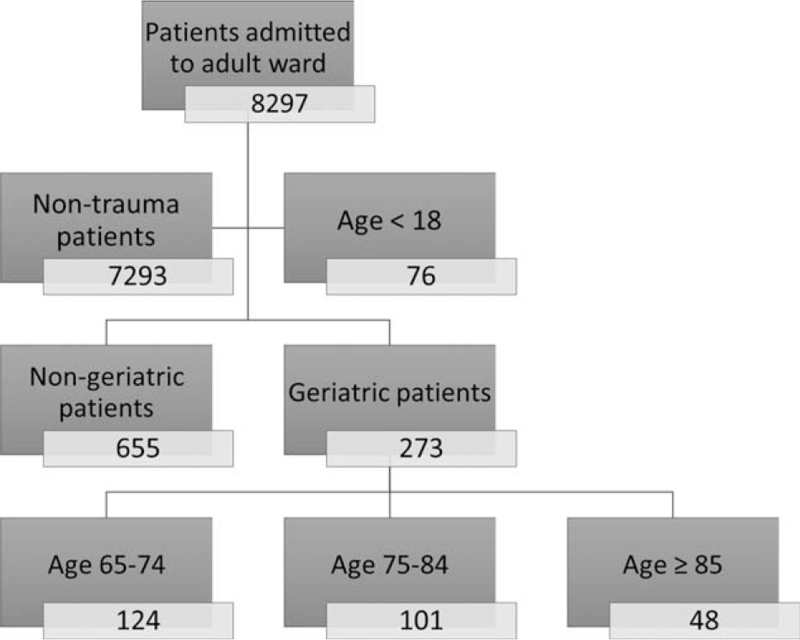
Study profile. The number in each box represents the number of cases for each group.

**Figure 2:**
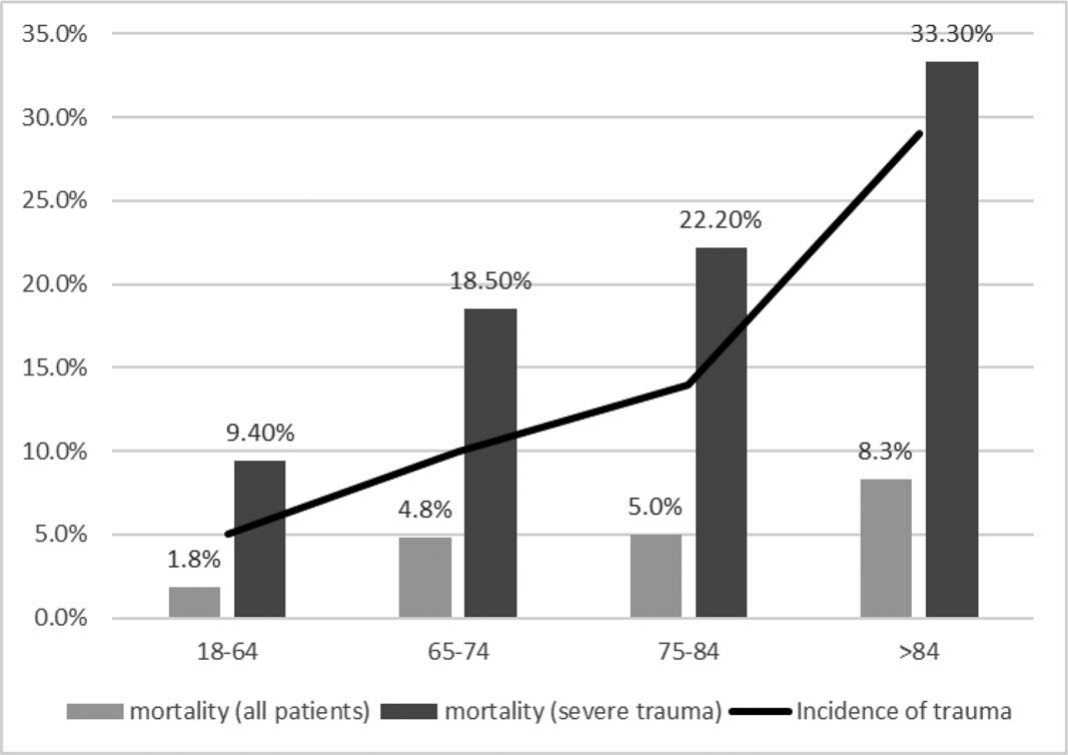
We discovered that the incidence of trauma increased with age (18–64 vs 65–74 vs 75–84 vs ≥ 85 years: 0.5/1000 vs 1.0/1000 vs 1.4/1000 vs 2.9/1000). Moreover, we observed an increase in mortality with age.

Figure 1 showed the study profile. In 2016, the number of citizens living in the city of study hospital was 1,886,033.^[18]^ We thus used citizens as the analysis unit to estimate the incidence of trauma in various age intervals. Of all hospitalized patients, the geriatric group had a higher incidence of trauma than did the nongeriatric group (1.1/1000 vs 0.5/1000). Additionally, we observed that the incidence of trauma increased with age (65–74 years vs 75–84 years vs ≥85 years: 1.0/1000 vs 1.4/1000 vs 2.9/1000). This incidence of trauma is illustrated in Figure 2.

Table 1 presents a comparison of all geriatric and nongeriatric patients with trauma in terms of demographic characteristics. Except for age, the geriatric and nongeriatric groups exhibited similar sex distributions and proportions of patients with a Charlson comorbidity index of >0. Regarding prehospital conditions (location of injury, mechanism of injury, and time of transportation from the site of injury to the hospital), we observed that the geriatric group was associated with a longer time of transportation from the site of injury to the ED compared with the nongeriatric group. Concerning vital signs and triage in the ED, we found no differences between the geriatric and nongeriatric groups, except for respiratory rate, which was lower in the geriatric group.

**Table 1:** Comparison of the demographic characteristics between geriatric and nongeriatric groups for all adult patients with trauma.

	Geriatric (All patients)	Non-geriatric (All patients)	*P*
Case number (%)	273 (29.4%)	655 (70.6%)	
Average age (yr)	76.3 ± 8.0	42.3 ± 14.4	.000
Sex (Male)	58.6%	59.8%	.726
Charlson Comorbidity Index >0	28.6%	25.5%	.333
Locations of injury			
Street	51.3%	49.5%	.492
Factory/Farm/Mine	15.0%	16.3%	
Home	12.8%	15.3%	
Public places	5.1%	3.1%	
Others/ Unknown	15.8%	15.3%	
Mechanisms of injury			
Traffic accident	49.5%	49.5%	.616
Fall	29.7%	33.4%	
Crush	7.7%	9.2%	
Burn/Electricity	5.9%	4.6%	
Penetrating injury	4.8%	3.5%	
Asphyxia/Drowning	1.8%	3.1%	
Suicide	0.7%	0,8%	
Prehospital transport			
Transport by EMTs	44.0%	49.6%	.107
Transport by themselves	30.4%	30.7%	
Transfer from other hospital	25.6%	19.7%	
Time from injury to ED (median, interquartile range, min)	47.0 (28.0–92.5)	42.0 (25.0–80.0)	.005
Vital signs at ED arrival			
GCS	14.3 ± 2.3	14.4 ± 2.0	.196
SAP (mm Hg)	145.5 ± 32.3	144.9 ± 32.9	.626
HR (beat/min)	85.1 ± 19.3	86.4 ± 19.2	.874
RR (respiration/min)	17.3 ± 2.9	17.4 ± 2.4	.022
Temperature (°C)	36.5 ± 0.6	36.5 ± 0.7	.497
Shock Index (HR/SAP)	0.6 ± 0.2	0.6 ± 0.2	.318
Triage			
Triage 1	7.7%	7.8%	.873
Triage 2	46.2%	44.4%	
Triage 3	45.8%	47.6%	
Triage 4	0.4%	0.2%	
Injury regions (Abbreviated Injury Scale >2)			
Head and neck	22.7%	20.9%	.114
Face	0.4%	1.1%	
Chest	7.7%	9.2%	
Abdomen	2.9%	2.9%	
Extremity	23.8%	27.8%	
External	1.8%	0.3%	
Severity of trauma			
RTS	7.6603 ± 0.7588	7.6868 ± 0.6582	.294
ISS	10.1 ± 10.7	9.2 ± 7.6	.004
NISS	12.7 ± 12.6	11.5 ± 10.5	.013
TRISS	0.9475 ± 0.1440	0.9571 ± 0.1251	.087
Laboratory tests			
White cell count (cell/uL)	11800 ± 4800	11400 ± 4600	.178
Hemoglobin (g/L)	13.6 ± 2.0	13.4 ± 2.2	.090
Platelet (cell/uL)	240700 ± 67700	242300 ± 71100	.882
PT (INR)	1.0 ± 0.1	1.0 ± 0.1	.518
APTT (sec)	25.9 ± 3.9	25.9 ± 3.3	.803
Sodium (mmol/L)	139.0 ± 3.0	139.5 ± 2.5	.738
Potassium (mmol/L)	3.8 ± 0.3	3.8 ± 0.4	.034
Glucose (g/L)	140.7 ± 62.1	135.1 ± 48.5	.012
Creatinine (g/L)	1.0 ± 0.9	1.0 ± 0.7	.734
Alanine transaminase (U/L)	32.0 ± 56.6	32.6 ± 47.5	.975
Management and hospital course			
Surgery	65.9%	70.7%	.000
Time from ED to ward (median, interquartile range, min)	40.5 (19.0–83.0)	40.5 (20.0–75.5)	.380
Time from ED to surgery (median, interquartile range, min)	323.5 (211.3–746.8)	347.0 (199.0–671.0)	.808
Hospital stay (day)	9.3 ± 10.9	8.2 ± 9.1	.457
ICU requirement	22.3%	16.9%	.054
ICU stay (day)	8.1 ± 12.6	7.5 ± 7.6	.115

^∗^SAP = systolic arterial pressure ^†^HR = heart rate ^‡^RR = respiratory rate ^§^RTS = revised trauma score ^¶^ISS = Injury Severity Score, jNISS = New Injury Severity Score, ^∗∗^TRISS = Trauma Injury Severity Score, jjGCS = Glasgow Coma Scale, ^∗∗∗^EMS: emergency medical service, ≠ICU = intensive care unit.

The geriatric and nongeriatric groups also exhibited similar distributions with respect to region of injury. The geriatric group had a higher Injury Severity Score and New Injury Severity Score than did the nongeriatric group. However, the geriatric and nongeriatric groups exhibited similar Revised Trauma Scores and Trauma Injury Severity Scores. The average values of all laboratory test parameters were within normal limits for the geriatric and nongeriatric groups, except for the average white blood cell count, which was elevated in these 2 groups. We observed significant differences in potassium and glucose levels between the geriatric and nongeriatric groups. Other laboratory test parameters were similar between the 2 groups.

Fewer geriatric patients underwent surgery during their hospitalizations than nongeriatric patients (65.9% vs 70.7%, *P* = .000). Other management procedures and hospital courses were similar between the geriatric and nongeriatric groups.

### 3.2 Patients with severe trauma injury severity score >15

Table 2 presents a comparison of geriatric and nongeriatric patients with severe trauma in terms of demographic characteristics. This subgroup analysis included 58 and 128 geriatric and nongeriatric patients, respectively. Except for age, the geriatric and nongeriatric groups exhibited similar sex distributions and percentages of patients with a Charlson comorbidity index of >0, we discovered that the geriatric group had a higher proportion of patients transferred from other hospitals than did the nongeriatric group.

**Table 2:** Comparison of demographic characteristics between geriatric and nongeriatric groups for patients with severe trauma (Injury Severity Score >15).

	Geriatric (ISS > 15 patients)	Non-geriatric (ISS > 15 patients)	*P*
Case number (%)	58 (31.2%)	128 (68.8%)	
Average age (yr)	76.1 ± 8.1	41.2 ± 14.5	.000
Sex (Male)	65.5%	65.5%	.989
Charlson Comorbidity Index > 0	34.5%	29.7%	.513
Locations of injury			
Street	70.7%	61.7%	.561
Factory/ Farm/Mine	6.9%	9.4%	
Home	12.1%	10.2%	
Public places	3.4%	3.9%	
Others/ Unknown	6.9%	14.8%	
Mechanisms of injury			
Traffic accident	69.0%	57.0%	.264
Fall	24.1%	30.5%	
Others	6.9%	12.5%	
Prehospital transport			
Transport by EMTs	51.7%	57.0%	.041
Transport by themselves	6.9%	17.2%	
Transfer from other hospital	41.4%	25.8%	
Time from injury to ED (median, interquartile range, min)	51.0 (26.5–164.0)	37.0 (25.0–112.0)	.334
Vital signs at ED arrival			
GCS	12.2 ± 4.3	12.8 ± 3.6	.219
SAP (mm Hg)	146.7 ± 40.4	139.8 ± 39.9	.891
HR (beat/min)	87.9 ± 24.3	90.6 ± 21.8	.611
RR (respiration/min)	17.1 ± 4.7	17.5 ± 3.5	.029
Temperature (°C)	36.5 ± 0.6	36.3 ± 0.9	.783
Shock Index (HR/SAP)	0.6 ± 0.3	0.7 ± 0.3	.363
Triage			
Triage 1	32.8%	29.7%	.395
Triage 2	55.2%	50.0%	
Triage 3	12.1%	20.3%	
Injury regions (Abbreviated Injury Scale >2)			
Head and neck	77.6%	81.3%	.188
Face	1.7%	3.9%	
Chest	25.9%	35.2%	
Abdomen	8.6%	7.0%	
Extremity	8.6%	16.4%	
External	3.4%	0.0%	
Severity of trauma			
RTS	7.0701 ± 1.4942	7.2367 ± 1.2989	.211
ISS	25.2 ± 14.6	21.8 ± 7.0	.000
NISS	32.2 ± 12.9	28.7 ± 9.7	.036
TRISS	0.8094 ± 0.2716	0.8687 ± 0.2168	.026
Laboratory tests			
White cell count (cell/uL)	13800 ± 6400	12500 ± 5400	.069
Hemoglobin (g/L)	13.2 ± 1.8	12.8 ± 2.4	.043
Platelet (cell/uL)	209800 ± 67800	233100 ± 98000	.324
PT (INR)	1.0 ± 0.1	1.0 ± 0.2	.671
APTT (sec)	27.2 ± 7.9	26.2 ± 5.9	.239
Sodium (mmol/L)	138.1 ± 4.2	139.5 ± 2.7	.466
Potassium (mmol/L)	3.7 ± 0.4	3.7 ± 0.5	.290
Glucose (g/L)	157.4 ± 60.1	153.7 ± 54.1	.705
Creatinine (g/L)	1.4 ± 2.1	1.0 ± 0.5	.002
Alanine transaminase (U/L)	56.4 ± 126.5	41.8 ± 43.6	.028
Management and hospital course			
Surgery	27.6%	44.5%	.028
Time from ED to ward (median, interquartile range, min)	66.0 (23.0–164.0)	56.0 (21.0–168.0)	.468
Time from ED to surgery (median, interquartile range, min)	408.5 (109.5–1552.3)	355.0 (94.5–1328.0)	.805
Hospital stay (day)	10.2 ± 10.2	8.9 ± 10.5	.931
ICU requirement	27.6%	16.4%	.077
ICU stay (day)	6.8 ± 9.6	5.3 ± 4.2	.166

^∗^SAP = systolic arterial pressure ^†^HR = heart rate ^‡^RR = respiratory rate ^§^RTS = Revised Trauma Score ^¶^ISS = Injury Severity Score, jNISS = New Injury Severity Score, ^∗∗^TRISS = Trauma Injury Severity Score, jjGCS = Glasgow Coma Scale, ^∗∗∗^EMS = emergency medical service, ≠ICU = intensive care unit.

We noted no differences between the geriatric and nongeriatric groups with respect to vital signs and triage in the ED except for respiratory rate, which was lower in the geriatric group.

Furthermore, the geriatric and nongeriatric groups exhibited similar distributions with respect to region of injury. The geriatric group had higher Injury Severity Score and New Injury Severity Score and lower Trauma Injury Severity Score than did the nongeriatric group. However, the 2 groups exhibited similar Revised Trauma Scores.

The values of 3 laboratory test parameters differed significantly between the geriatric and nongeriatric groups. Specifically, the geriatric group had higher levels of hemoglobin, creatinine, and alanine transaminase.

Fewer geriatric patients underwent surgery than did nongeriatric patients (27.6% vs 44.5%, *P* = .028). Other managements and hospital courses were similar between the geriatric and nongeriatric groups.

### 3.3 Prognosis

**Figure 3:**
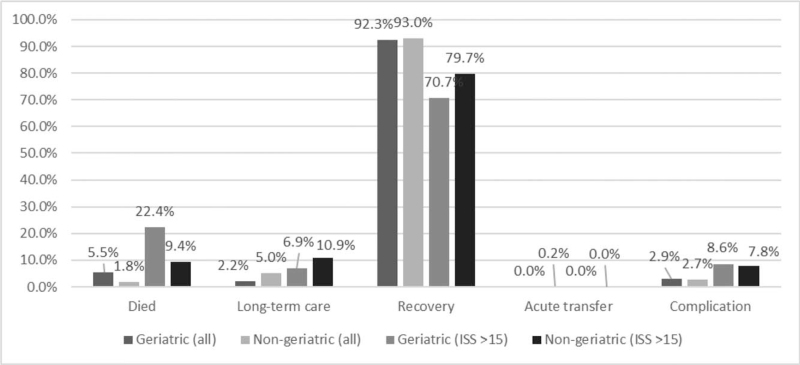
Regarding prognosis of the geriatric and nongeriatric groups, the geriatric group had a higher proportion of patients who died and lower proportion of patients who required long-term care. For all patients and for patients with severe trauma, the incidence rates of complications were comparable between geriatric and nongeriatric groups.

Figure 3 summarizes the prognosis of all patients. We observed an increase in mortality with age (all patients with trauma: 18–64 years vs. 65–74 years vs. 75–84 years vs ≥85 years: 1.8% vs 4.8% vs 5.0% vs 8.3%; patients with severe trauma: 18–64 years vs 65–74 years vs 75–84 years vs ≥85 years: 9.4% vs 18.5% vs 22.2% vs 33.3%). Figure 3 illustrates the observed incidence and mortality in different age groups in our study populations.

Comparing the geriatric and nongeriatric groups, the geriatric group had a higher mortality rate and less need for long-term care (mortality: 5.5% vs 1.8%; long-term care: 2.2% vs 5.0%; recovery: 92.3% vs 93.0%; acute transfer: 0.0% vs 0.2%, *P* = .005). The difference in mortality between the geriatric and nongeriatric group was greater in severe trauma (22.4% vs 9.4%, *P* = .046). Regarding complication rates, geriatric patients and nongeriatric patients in this study showed similar complication rates.

### 3.4 Patients who underwent surgery

We discovered that the geriatric trauma patients had a lower surgical rate compared to nongeriatric patients; such difference was greater in severe trauma cases and might contributed to the prognostic differences between the 2 groups. Therefore, we selected all patients who had undergone surgery and divided them into geriatric (184 patients) and nongeriatric (465 patients) groups to compare their trauma severity, resource requirements, and prognosis (Table 3). The geriatric group had a lower percentage of patients with multiple trauma (defined as patients with an Injury Severity Score of >2 in 2 regions) than did the nongeriatric group; however, the difference did not reach statistical significance (geriatric group vs nongeriatric group: 3.8% vs 7.5%, *P* = .082). Furthermore, the geriatric group included fewer patients who sustained severe injuries in the head and neck regions (defined as an Abbreviated Injury Scale >2 in the head and neck regions) than did the nongeriatric group (20.0% vs 42.3%, *P* = .000). The need for intensive care and the need for life-saving procedures were comparable between the geriatric and nongeriatric groups; nevertheless, the geriatric group had higher proportions of patients who died, required long-term care, and had complications than did the nongeriatric group (mortality: 3.8% vs 1.3%; long-term care: 9.8% vs 1.7%; recovery: 85.9% vs 95.9%, *P* = .000; complication: 3.8% vs 3.2%, *P* = .000).

**Table 3:** Data for all patients who underwent surgery. We observed that the geriatric group had a lower percentage of patients with multiple trauma than did the nongeriatric group, although the difference was nonsignificant. The geriatric group included fewer patients who sustained severe injuries in the head and neck regions (defined as Abbreviated Injury Scale of >2 for head and neck regions). Nevertheless, the geriatric group had a higher proportion of patients who died, required log-term care, and had complications.

	Geriatric (184)	Nongeriatric (465)	*P*
Multiple trauma^†^	3.8%	7.5%	.082
Head and neck AIS^‡^ > 2	20.0%	42.3%	.000
Requirement for intensive care	11.4%	11.2%	.933
Life-saving procedures^∗^	4.3%	7.1%	.194
Died	3.8%	1.3%	.000
Long-term care	9.8%	1.7%	
Recovery	85.9%	95.9%	
Acute transfer	0.5%	1.1%	
Complications	3.8%	3.2%	.000

† Patients whose Injury Severity Score was >2 in two regions.

‡ Abbreviated Injury Scale.

∗ Included emergent life-saving surgery and transarterial embolization.

## 4 Discussion

From the results, we found the incidence and mortality rate of trauma increased with age in geriatric group. Compared to the nongeriatric group, the geriatric group showed higher mortality rate, fewer patients underwent surgical treatment, higher trauma scores, a longer time of transportation from the site of injury to the ED, more requirements for inter-hospital transfer, and clinically insignificant differences in vital signs and laboratory tests.

A survey in the United States for the period 2000 to 2011 revealed that the mean age of inpatients with trauma increased between 2000 and 2011.^[19]^ The aging of the inpatient trauma population is a reflection of population aging in most countries worldwide. The findings of this study support that the incidence of inpatient trauma increased with age. Moreover, we observed that an increase in the incidence of trauma was accompanied by an increase in mortality in geriatric patients. Aging predisposes the geriatric population to trauma and increases the risk of them requiring care.

In the prehospital settings, we discovered that more than 40% of geriatric patients with severe trauma were transferred from other hospitals. Chi-Mei Medical Center is a regional level I trauma center in southern Taiwan and usually takes over difficult patients as well as patients with severe trauma transferred from other hospitals. For transfer patients, the time from injury to the ED (i.e., ED at the receiving hospital) was usually longer because it included the time spent in the referring hospital. Therefore, we considered that the longer time from injury to ED in geriatric trauma patients was due to the higher percentage of patient transfer in the geriatric group.

Regarding triage, vital signs in ED, and laboratory test parameters, the geriatric and nongeriatric groups differed with respect to respiratory rate and potassium, glucose, hemoglobin, creatinine, and alanine transaminase levels. However, for most variables that differed significantly between the groups, the data were within or near normal limits. Such slight differences seldom change the decision making of trauma surgeons in patient management. Scholars commonly assume that the vital signs of geriatric patients might be altered by medications or that the laboratory parameters of such patients might be within abnormal limits; however, we did not observe meaningful differences in triage, vital signs in ED, or laboratory test parameters between the geriatric and nongeriatric patients.

Regarding various factors from prehospital settings to hospital management, we observed that the differences between the geriatric and nongeriatric groups were negligible. Aging is a definite predictor of poor outcomes for patients with trauma. For all patients with trauma and for patients with severe trauma, we noted that the geriatric group had a higher mortality rate than the nongeriatric group. Three possible explanations for these findings are outlined as follows:

1. Under the same mechanism of injury, geriatric patients incurred more severe trauma than younger patients.
2. For a certain injury severity level, geriatric trauma patients had higher mortality rates than younger patients because of their existing comorbidities and limited physiological reserves.
3. Finally, geriatric patients undergo less invasive interventions during hospitalization than their younger counterparts.

The first 2 explanations are consistent with the finding of Evans et al, who revealed that even minor trauma, such as ground-level falls, could result in severe injury and sequelae in geriatric patients.^[20]^ Frailty, sarcopenia, and polypharmacy (especially anticoagulants and antiplatelets) are conducive to poor outcomes in elderly patients.^[5]^

With recent advancements in palliative care services, increasing numbers of patients and their families opt for palliative care over aggressive treatment for severely ill patients.^[21]^ Older individuals exhibit poorer outcomes than their younger counterparts, as indicated by their higher in-hospital mortality rates, accelerated mortality following discharge, and—for those who survive—worse functional outcome at 6 months.^[22]^ Hence, increasing numbers of geriatric patients and their families prefer tailoring the goals of treatment. This approach reduces the burden induced by long-term care and improves the quality of life of patients. We also observed that fewer patients in the geriatric group underwent surgery than those in the nongeriatric group. Among patients who underwent surgery, the geriatric group had a lower percentage of patients with multiple trauma or severe head and neck injuries, because polytrauma and severe head injury are often resulted in long-term disability of the trauma victims.^[2]^ These findings indicate that elderly patients are prone to select noninvasive management, particularly the severely injured patients or patients with injuries that might result in long-term disability. Thus, the preference of older individuals to avoid aggressive treatment might also contributes to their higher mortality and less need for long-term care.

### 4.1 Limitations

This study has several limitations. First, the data were derived from a single trauma center, which may not be representative of the actual geriatric trauma population. In addition, the estimated incidence of trauma may be inaccurate. Nevertheless, the study hospital is a regional trauma center and has the most ED visits (120,000 annually) among all hospitals in the region. We believe that the collected data could portray the actual situation of geriatric trauma in Taiwan. Second, because all the data were obtained from retrospective chart reviews, the preinjury condition of each patient was not recorded precisely. Some elderly individuals may had been referred from convalescent care centers before their admission. They would be sent back for long-term care even if the injury did not result in further disability. Therefore, the need for long-term care might be overestimated in this study. However, if the need for long-term care were reduced, the difference between the geriatric and nongeriatric groups would be more pronounced than that presented herein. Third, the study period is pretty much short for such an investigation, a further study which includes a longer period would demonstrate a clearer picture of this issue. Lastly, we did not perform follow-up assessments to verify the condition of each patient after discharge. Some patients might have recovered completely after undergoing care in convalescent care centers. We agree that this study only demonstrated the acute differences between the geriatric and nongeriatric populations following injury. Future studies are required to understand the long-term results of geriatric trauma.

## 5 Conclusion

We observed that an increase in the incidence of trauma was accompanied by an increase in mortality in geriatric patients. Under similar covariants from prehospital settings to hospital management, the geriatric patients incurred more severe injuries and had higher mortality rates than nongeriatric patients. Aging is definitely a predictor of poor outcomes for patients with trauma. Limited physiological reserves and the preference for less aggressive treatment could be the main reasons for poor outcomes in elderly individuals.

## Author contributions

**Conceptualization:** Nan-Chun Wu, Chien-Chin Hsu, Kuo-Tai Chen.

**Data curation:** Pei-Chen Lin, Hsiu-Chen Su.

**Formal analysis:** Chien-Chin Hsu.

**Methodology:** Pei-Chen Lin, Chien-Chin Hsu.

**Project administration:** Nan-Chun Wu.

**Resources:** Hsiu-Chen Su.

**Software:** Pei-Chen Lin.

**Supervision:** Nan-Chun Wu.

**Validation:** Hsiu-Chen Su.

**Visualization:** Kuo-Tai Chen.

**Writing – original draft:** Pei-Chen Lin.

**Writing – review & editing:** Kuo-Tai Chen.
